# Regular proton pump inhibitor use and incident dementia: population-based cohort study

**DOI:** 10.1186/s12916-022-02478-y

**Published:** 2022-09-01

**Authors:** Peidong Zhang, Zhihao Li, Peiliang Chen, Ao Zhang, Yu Zeng, Xiru Zhang, Qingmei Huang, Dan Liu, Songtao Qi, Chen Mao

**Affiliations:** 1 Department of Epidemiology, School of Public Health, Southern Medical University, Guangzhou, 510515 Guangdong China; 2 Department of Neurosurgery, Nanfang Hospital, Southern Medical University, Guangzhou, 510515 Guangdong China; 3 State Key Laboratory of Molecular Neuroscience and Center of Systems Biology and Human Health, Division of Life Science, Hong Kong University of Science and Technology, Hong Kong, China; 4 Guangdong Provincial Key Laboratory of Bone and Joint Degeneration Diseases, Department of Cell Biology, School of Basic Medical Sciences, Southern Medical University, Guangzhou, Guangdong China; 5 Department of Public Health and Preventive Medicine, School of Medicine, Jinan University, Guangzhou, Guangdong China; 6 Laboratory for Precision Neurosurgery, Nanfang Hospital, Southern Medical University, Guangzhou, Guangdong China; 7 Microbiome Medicine Center, Department of Laboratory Medicine, Zhujiang Hospital, Southern Medical University, Guangzhou, Guangdong China

**Keywords:** Proton pump inhibitors, Dementia, Alzheimer’s disease, APOE, Amyloid-β

## Abstract

**Background:**

To examine the association between regular use of proton pump inhibitors and the risk of incident dementia, including dementia subtypes, and whether the association differs between *APOE* genotypes.

**Methods:**

Based on a prospective analysis of data from the UK Biobank, 501,002 individuals (female, 54.4%) aged between 40 and 70 years, who had no prevalent dementia at baseline, were enrolled between 2006 and 2010 and followed up to 2018. We compared all-cause dementia, Alzheimer’s disease (AD), and vascular dementia (VaD) incidence rates between proton pump inhibitor users and non-users by the Cox proportional hazard model.

**Results:**

During 4,438,839 person-years of follow-up (median length of follow-up, 9.0 years), there were 2505 incident cases of all-cause dementia, including 932 cases of AD and 524 cases of VaD. The incident rate of all-cause dementia among proton pump inhibitor users was 1.06 events per 1000 person-years, compared with 0.51 events per 1000 person-years among non-users. After adjustment for multiple confounders and indications, the hazard ratios (HRs) of the proton pump inhibitor users were 1.20 (95% CI, 1.07–1.35) for incident all-cause dementia, 1.23 (95% CI, 1.02–1.49) for incident AD, and 1.32 (95% CI, 1.05–1.67) for incident VaD. In addition, the association between proton pump inhibitor use and all-cause dementia differed by *APOE* genotype (*P* for interaction = 0.048). Among *APOE* ε4 heterozygotes, the fully adjusted HR of proton pump inhibitor use was 1.46 (95% CI, 1.22–1.75) and 1.68 (95% CI, 1.36–2.07), especially for individuals aged 65 years and older.

**Conclusions:**

The finding of this large population-based cohort study indicates that the use of proton pump inhibitors is associated with an increased risk of incident dementia, particularly among *APOE* ε4 heterozygotes.

**Supplementary Information:**

The online version contains supplementary material available at 10.1186/s12916-022-02478-y.

## Background

Proton pump inhibitors (PPIs) are widely used in the treatment of gastric acid-related disorders such as peptic ulcer disease, gastroesophageal reflux disease (GERD), non-steroidal anti-inflammatory drug (NSAID)-associated ulcers, and eradication of *Helicobacter pylori* [1]. In many countries, including the UK, some PPIs are available for over-the-counter purchase, which increases public accessibility. Furthermore, PPIs are often prescribed in and out of the hospital for incorrect indications or long-term use that does not meet guidelines [2, 3]. With the increasing use of PPIs [4], more attention has been paid to the research on its side effects [5]. A series of studies have reported associations between PPI use and cardiovascular disease [6, 7], fracture [8], kidney disease [9], infections [10], and diabetes [11], but the association with dementia is controversial [12].

Dementia is characterized by inexorably progressive impairment in cognitive and independent living functions. Alzheimer’s disease (AD), vascular dementia (VaD), Lewy body, and frontotemporal dementia are the most common pathologies. It is estimated that there were 35.6 million dementia patients worldwide in 2010, and the number may reach 65.7 million in 2030 [13]. Meanwhile, the worldwide costs of dementia were estimated at $818 billion in 2015 [14]. To prevent dementia, reducing risk factor exposure is vital in the circumstance of limited treatment. Several cohort studies reported the association between PPI use and all-cause dementia or AD among the elderly, and the hazard ratios (HRs) of PPI users were 1.38 to 1.44 [15, 16]. However, other studies showed conflicting conclusions, and most of these studies did not observe any associations [17–22]. Therefore, the association between PPI use and dementia is still uncertain.

PPIs are aimed to reduce the gastric acid secretion of the parietal cell by inhibiting (H(+), K(+))-ATPase [23]. Similar enzymes are also found in microglia lysosomes [24], and the lysosomal acidic environment is essential for amyloid-β (Aβ) clearance, the disorder of which may lead to neurodegeneration and dementia [25]. A study reveals that PPIs may increase Aβ deposition in the mouse brain by affecting the β- and γ-secretases [26]. However, precision measurement of Aβ metabolism in a large population would be difficult. A measurable proxy for Aβ is required to infer whether the PPIs promote dementia via affecting Aβ metabolism.

Apolipoprotein E (Apo-E) is a primary cholesterol carrier involved in lipid transport, and *APOE* ε4 alleles are the main genetic risk factors for AD and dementia due to their reduced capacity for Aβ transport [27]. *APOE* ε4 may also promote AD by reducing the ability of astrocytes to remove toxic fatty acids from the extracellular milieu [28]. Possible mechanisms for the potential association between PPIs and dementia and whether PPIs can interact with *APOE* require evidence from population-based studies.

To further explicitly whether regular PPI use is associated with incident all-cause dementia and pathological specific dementia (AD and VaD), we conducted a large prospective cohort study in the UK Biobank. Furthermore, we also tried to explore the differences in the associations among different *APOE* ε4 genotypes, a potential regulatory gene of Aβ metabolism, to suggest biological mechanisms.

## Results

### Participant characteristics

**Fig. 1:**
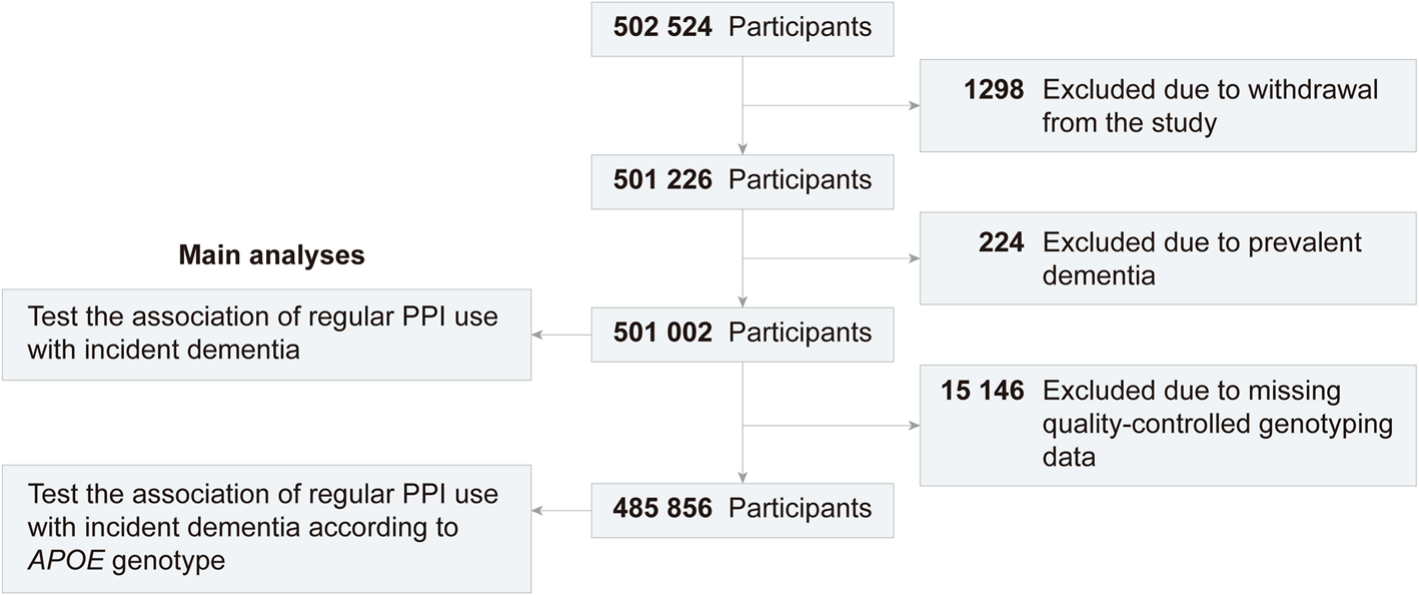
Flowchart of participant enrolment. Abbreviations: PPI, proton pump inhibitor; *APOE*, apolipoprotein E

Table 1 presents the baseline characteristics of eventually included participants stratified by PPI users or non-users. Of the 501,002 individuals (mean [SD] age, 56.5 [8.1] years), 272,605 (54.4%) were female and 53,735 (10.7%) were regular PPI users (Fig. 1). The regular users were slightly older, had higher BMIs, more smoking exposure, less alcohol consumption, and more comorbidity and regular drug use.

**Table 1:** Baseline characteristics

Characteristics	No. (%)^a^	
	PPI non-users	PPI users
Total	447,267	53,735
Age (years), mean (SD)	56.2 (8.1)	59.4 (7.3)
Sex		
Female	243,253 (54.4)	29,352 (54.6)
Male	204,014 (45.6)	24,383 (45.4)
Ethnicity		
White	422,581 (94.5)	51,240 (95.4)
Others	24,686 (5.5)	2495 (4.6)
Education		
Higher	220,614 (49.3)	20,767 (38.6)
Upper secondary	39,176 (8.8)	3660 (6.8)
Lower secondary	92,519 (20.7)	11,203 (20.8)
Vocational	22,670 (5.1)	3563 (6.6)
Others	72,288 (16.2)	14,542 (27.1)
Household income (£)		
<18,000	99,929 (22.3)	18,700 (34.8)
18,000–30,999	114,099 (25.5)	14,956 (27.8)
31,000–51,999	117,645 (26.3)	11,560 (21.5)
52,000–100,000	91,382 (20.4)	6918 (12.9)
>100,000	24,212 (5.4)	1601 (3.0)
Townsend deprivation index, median [interquartile range]	−2.2 [−3.7, 0.5]	−1.8 [−3.5, 1.2]
Body mass index (kg/m^2^), mean (SD)	27.2 (4.7)	29.1 (5.1)
Regular physical activity		
No	185,029 (41.4)	25,213 (46.9)
Yes	262,238 (58.6)	28,522 (53.1)
Smoking status		
Never	249,076 (55.7)	25,226 (46.9)
Former	150,929 (33.7)	22,604 (42.1)
Current	47,262 (10.6)	5905 (11.0)
Alcohol consumption (g/day)		
0	107,342 (24.0)	17,191 (32.0)
0.01–13.99	167,958 (37.6)	18,331 (34.1)
14–27.99	96,157 (21.5)	9685 (18.0)
≥28	75,810 (16.9)	8528 (15.9)
Occupational exposure		
Rarely/never	355,224 (79.4)	43,967 (81.8)
Sometimes	57,614 (12.9)	5383 (10.0)
Often	34,429 (7.7)	4385 (8.2)
Health conditions		
Hypertension	110,673 (24.7)	22,237 (41.4)
Coronary heart disease	16,831 (3.8)	7377 (13.7)
Diabetes	20,061 (4.5)	5362 (10.0)
High cholesterol	49,626 (11.1)	11,814 (22.0)
Stroke	5395 (1.2)	1802 (3.4)
Traumatic brain injury	1393 (0.3)	222 (0.4)
Depression	22,812 (5.1)	5294 (9.9)
Anxiety	5539 (1.2)	1160 (2.2)
Sleep apnea	1240 (0.3)	368 (0.7)
Cancer	33,592 (7.5)	5739 (10.7)
GERD	5183 (1.2)	15,758 (29.3)
Barrett’s esophagus	237 (0.1)	1231 (2.3)
Gastroduodenal ulcer	2765 (0.6)	2840 (5.3)
Regular use of supplement or drugs		
Statin	68,147 (15.2)	18,474 (34.4)
Antihypertensive drugs	83,931 (18.8)	19,782 (36.8)
Anticholinergic drugs	38,032 (8.5)	11,747 (21.9)
Benzodiazepines	2424 (0.5)	1013 (1.9)
z-Hypnotics	1458 (0.3)	611 (1.1)
Aspirin	56,812 (12.7)	12,648 (23.5)
Non-aspirin NSAIDs	128,374 (28.7)	20,366 (37.9)
Multivitamin	139,534 (31.2)	17,859 (33.2)
H2RAs	7533 (1.7)	1921 (3.6)
*APOE* genotype		
*APOE* ε4 −/−	310,111 (71.5)	37,676 (72.3)
*APOE* ε4 +/−	113,253 (26.1)	13,280 (25.5)
*APOE* ε4 +/+	10,367 (2.4)	1169 (2.2)

*Abbreviations*: *PPI* proton pump inhibitor, *SD* standard deviation, *GERD* gastroesophageal reflux disease, *NSAIDs* non-steroidal anti-inflammatory drugs, *H2RAs* H2 receptor antagonists, *APOE* apolipoprotein E

^a^All variables globally significantly different between groups at *P* < 0.001, except for sex (*P* = 0.299)

### Associations of PPI use with dementia outcomes

**Fig. 2:**
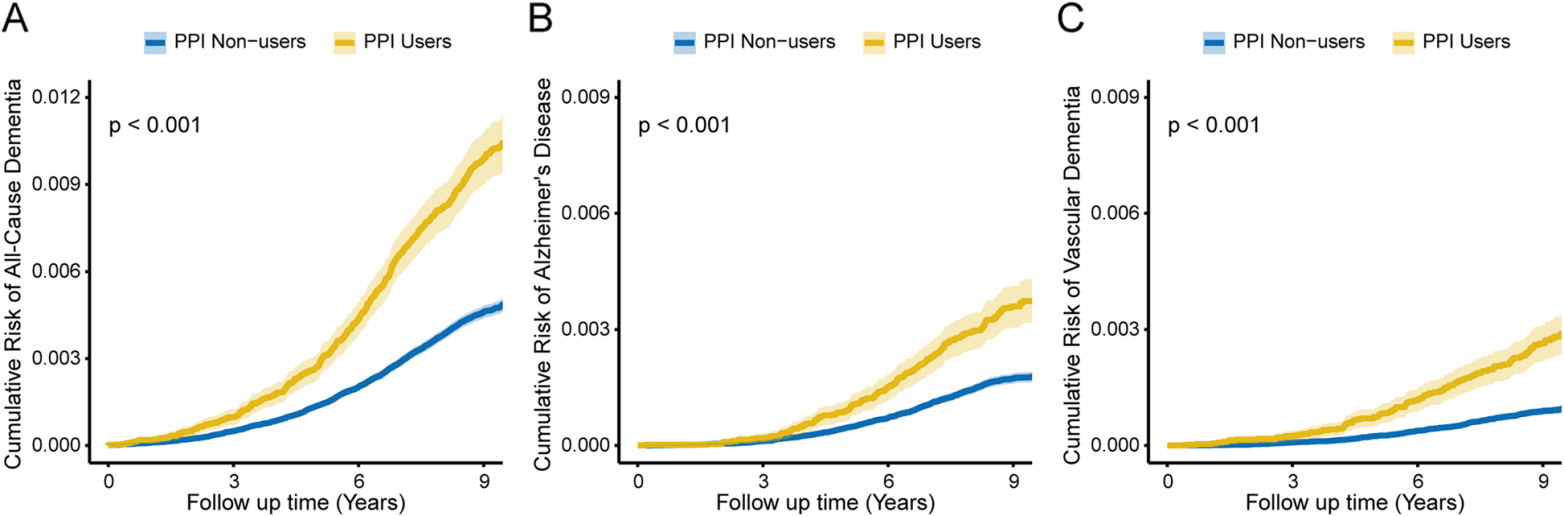
The cumulative risk of incident all-cause dementia (**A**), Alzheimer’s disease (**B**), and vascular dementia (**C**) according to regular PPI use. Abbreviation: PPI, proton pump inhibitor

Over 4,438,839 person-years of follow-up (median [interquartile range] length of follow-up, 9.0 [8.3–9.5] years), there were 2505 incident cases of all-cause dementia, including 932 cases of AD and 524 cases of VaD. The incident rate of all-cause dementia among PPI users was 1.06 events per 1000 person-years, compared with 0.51 events per 1000 person-years among non-users. The basic multivariable models found significant associations between PPI use and increased all-cause and cause-specific dementia risks (Table 2). After additional adjustment for clinical indications, the HRs of the PPI users were 1.20 (95% confidence interval [CI], 1.07–1.35; *P* = 0.001) for incident all-cause dementia, 1.23 (95% CI, 1.02–1.49; *P* = 0.031) for incident AD, and 1.32 (95% CI, 1.05–1.67; *P* = 0.017) for incident VaD. Figure 2 shows the cumulative risk of incident all-cause and cause-specific dementia in each PPI use status during follow-up (all *P* < 0.001).

**Table 2:** Associations of regular PPI use with incident dementia

Outcomes	PPI non-users (*n* = 447,267)	PPI users (*n* = 53,735)	Model 1^a^		Model 2^b^		Model 3^c^	
	No. of events (%)	No. of events (%)	HR (95% CI)	*P*-value	HR (95% CI)	*P*-value	HR (95% CI)	*P*-value
All-cause dementia	2008 (0.45)	497 (0.92)	1.49 (1.35–1.65)	<0.001	1.17 (1.05–1.29)	0.003	1.20 (1.07–1.35)	0.001
Alzheimer’s disease	752 (0.17)	180 (0.33)	1.41 (1.20–1.66)	<0.001	1.19 (1.00–1.41)	0.045	1.23 (1.02–1.49)	0.031
Vascular dementia	392 (0.09)	132 (0.25)	1.99 (1.63–2.42)	<0.001	1.32 (1.07–1.62)	0.009	1.32 (1.05–1.67)	0.017

*Abbreviations*: *PPI* proton pump inhibitor, *HR* hazard ratio, *CI* confidence interval

^a^Model 1: Cox proportional hazards regression adjusted for age and sex

^b^Model 2: Cox proportional hazards regression adjusted for model 1 and ethnicity, education, household income, Townsend deprivation index, smoking status, alcohol consumption, physical activity, BMI, occupational exposure, hypertension, coronary heart disease, diabetes, high cholesterol, stroke, traumatic brain injury, depression, anxiety, sleep apnea, cancer, and regular use of medications (statin, antihypertensive drugs, anticholinergic drugs, benzodiazepines, z-hypnotics, aspirin, non-aspirin NSAIDs, and multivitamin)

^c^Model 3: Cox proportional hazards regression adjusted for model 2 and GERD, Barrett’s esophagus, gastroduodenal ulcer, and regular H2RAs use

### Subgroup analyses

**Fig. 3:**
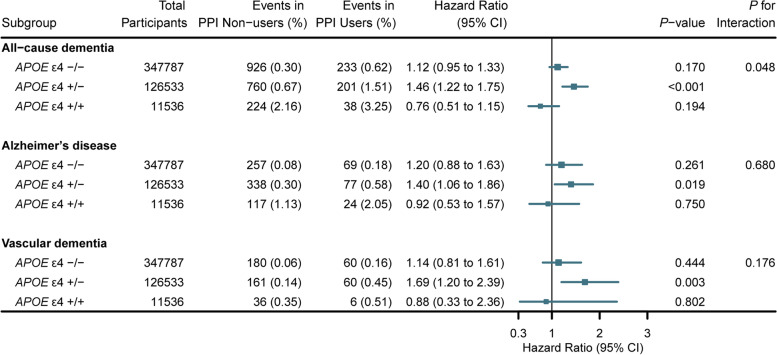
Association of regular PPI use with incident dementia stratified by *APOE* genotype. The vertical line indicates the reference value of 1. Estimated effects were based on the fully adjusted model. Abbreviations: PPI, proton pump inhibitor; *APOE*, apolipoprotein E; HR, hazard ratio; CI, confidence interval

To verify whether the *APOE* gene played a role as an effect modifier in the PPI use and dementia associations, we conducted subgroup analysis in different *APOE* ε4 genotypes by the fully adjusted model and tested the interactions. The association between PPI use and incident all-cause dementia was observed particularly among the *APOE* ε4 heterozygous (+/−) population (HR, 1.46; 95% CI, 1.22–1.75; *P* < 0.001), and the interaction was statistically significant (*P* for interaction = 0.048; Fig. 3). Additional file 1: Fig. S1 shows the cumulative risk of incident dementia in each PPI use status among different *APOE* ε4 genotype groups, and Additional file 1: Fig. S2 shows the combined effects of PPI use and *APOE* ε4 on the risk of dementia.

**Fig. 4:**
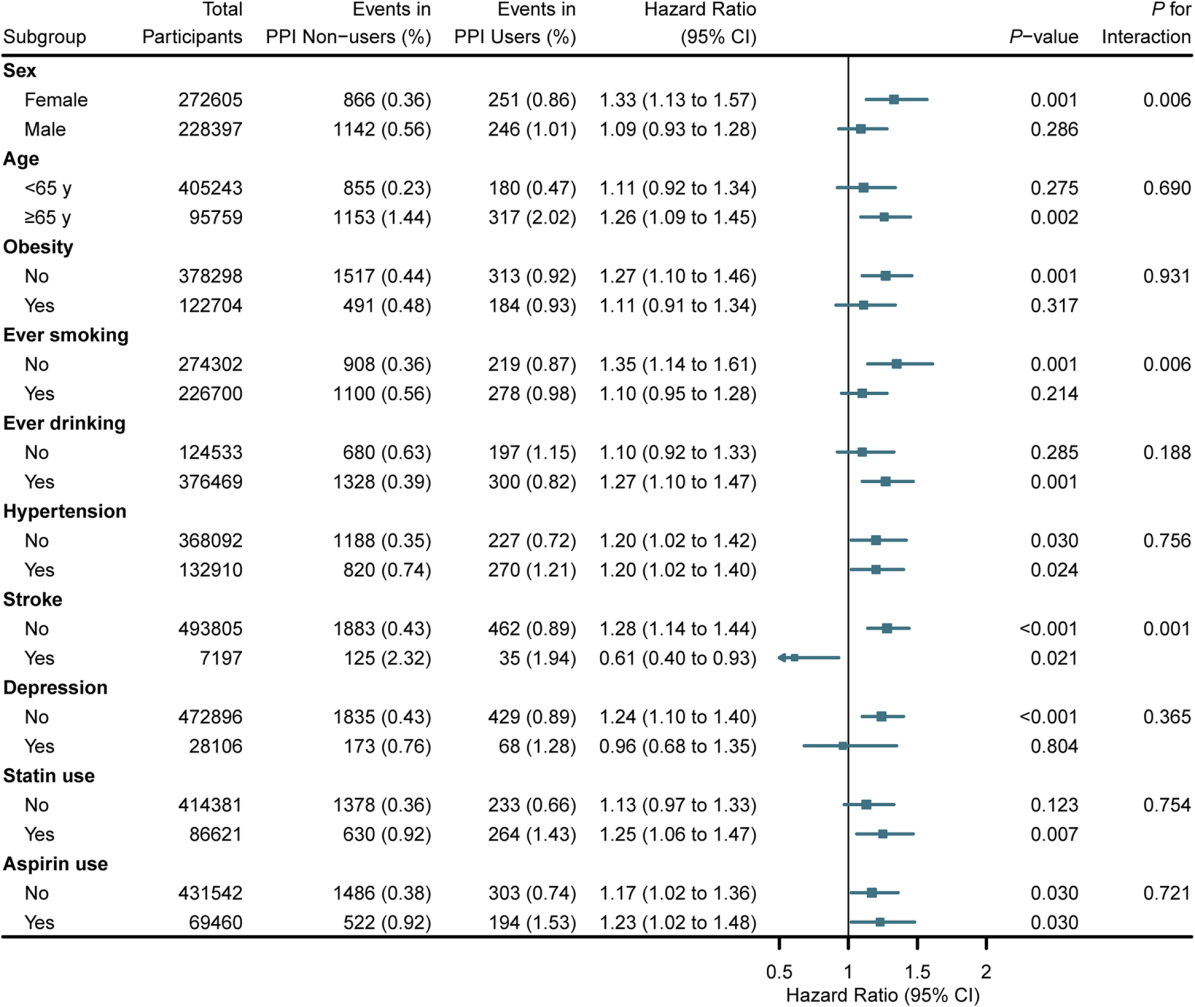
Association of regular PPI use with incident all-cause dementia stratified by potential risk factors. The vertical line indicates the reference value of 1. Estimated effects were based on the fully adjusted model. Abbreviations: PPI, proton pump inhibitor; HR, hazard ratio; CI, confidence interval

Furthermore, we conducted subgroup analyses according to other potential modifying factors. Regular PPI use and all-cause dementia associations were stronger among females, never smokers, and participants without stroke (all *P* for interaction < 0.05; Fig. 4). The combined effects of PPI use and significant modifying factors are shown in Additional file 1: Fig. S3. In addition, the associations of regular PPI use with cause-specific dementia were strong among females for AD and participants without stroke for VaD (all *P* for interaction < 0.05; Additional file 1: Fig. S4, S5). When each PPI was analyzed separately (Additional file 1: Table S3), the associations with all-cause dementia persisted in lansoprazole (HR, 1.26; 95% CI, 1.07–1.48; *P* = 0.007).

### Sensitivity analyses

We excluded participants younger than 65 years at baseline for sensitivity analysis to verify the effect modifier role of *APOE* ε4. The results showed that the interaction between PPI use and *APOE* ε4 genotype was still present for all-cause dementia (*P* for interaction = 0.012; Additional file 1: Fig. S6), and the HR of PPI users among *APOE* ε4 heterozygote was 1.68 (95% CI, 1.36–2.07; *P* < 0.001). Also, in the *APOE* ε4 heterozygotes, the HRs of PPI users were 1.55 (95% CI, 1.12–2.15; *P* = 0.008) and 1.80 (95% CI, 1.21–2.68; *P* = 0.004) for incident AD and VaD, respectively. However, interaction tests did not reach the statistically significant level (*P* for interaction > 0.05).

Results showed no significant change in PPI use and incident dementia associations when we excluded participants who developed dementia outcomes within the first 2 years of follow-up (Additional file 1: Table S4), excluded participants with missing values for covariates (Additional file 1: Table S5), and excluded participants who developed outcomes that were recorded on the death register data only (Additional file 1: Table S6). We included all covariates and used logistic regression to construct propensity scores with a *c*-statistic of 0.815 (95% CI, 0.813–0.817). The propensity score matching analysis results were consistent with the main model (Table 3).

**Table 3:** Associations of regular PPI use with incident dementia in propensity score matching model

Outcomes	PPI non-users (*n* = 107,470)	PPI users (*n* = 53,735)	PSM model 3^a^	
	No. of events (%)	No. of events (%)	HR (95% CI)	*P*-value
All-cause dementia	907 (0.84)	497 (0.92)	1.22 (1.08–1.37)	0.001
Alzheimer’s disease	321 (0.30)	180 (0.33)	1.25 (1.02–1.52)	0.028
Vascular dementia	223 (0.21)	132 (0.25)	1.30 (1.03–1.65)	0.028

*Abbreviations*: *PPI* proton pump inhibitor, *PSM* propensity score matching, HR hazard ratio, *CI* confidence interval

^a^Propensity scores were derived from logistic regression, which included age, sex, ethnicity, education, household income, Townsend deprivation index, smoking status, alcohol consumption, physical activity, BMI, occupational exposure, hypertension, coronary heart disease, diabetes, high cholesterol, stroke, traumatic brain injury, depression, anxiety, sleep apnea, cancer, GERD, Barrett’s esophagus, gastroduodenal ulcer, and regular use of medications (statin, antihypertensive drugs, anticholinergic drugs, benzodiazepines, z-hypnotics, aspirin, non-aspirin NSAIDs, multivitamin, and H2RA use)

## Discussion

In this population-based prospective cohort study of half a million participants, we found that regular PPI use was associated with an increased risk of incident all-cause dementia, AD, and VaD. Meanwhile, we found an interaction between PPI use and *APOE* ε4 genotype for all-cause dementia, and the association was more significant among *APOE* ε4 heterozygotes.

Our results were consistent with the previous studies that reported the association between PPI use and increased risk of dementia [15, 16, 29]. The study, which followed 70,000 participants over 75 years of age for 7 years, showed that PPI users had 1.44 times the risk of incident dementia as non-users [16]. Another study of more than 15,000 participants over 40 years of age without prevalent dementia followed for 8.44 years found that the adjusted HR for PPI users was 1.22 [29]. However, a separate series of observational studies reported that the associations were absent [19–22]. For example, a prospective study including over 70,000 participants showed that PPI use was not associated with dementia [21]. A systematic review and meta-analysis pooling 11 observational studies did not observe the association between short-term PPI use and dementia [30]. After that, the current study of more than 500,000 participants suggests that significant associations with incident dementia were still emerging with regular PPI use after adjusting for a wide range of lifestyle, comorbidity, and clinical indications. To our knowledge, this is the most extensive prospective study of PPI-dementia associations in the general population while providing some validation of the possible biological mechanisms of the association. Therefore, this study offers high-quality population-based evidence to assess the side effects accompanying regular PPI use.

Aβ aggregation to form plaques triggers neuronal dysfunction and death in the brain, which is the critical pathological feature of AD [31]. Studies of mouse models showed that PPIs might cross the blood-brain barrier [32, 33] and exacerbate Aβ production [26] to promote the development of dementia. Another mechanism was that the PPIs increase the accumulation of fibrillar Aβ by inhibiting the acidification of the degradation process in microglia [34, 35]. Aβ clearance from the brain requires the involvement of membrane cholesterol, and glial-derived *APOE* is a critical cholesterol transporter in the brain [31]. *APOE* ε4 is a determining risk factor of AD by promoting Aβ aggregation, associated with a 4-fold increased risk for a single allele [36]. We unprecedentedly reported the interaction between PPI use and *APOE* ε4 genotype in dementia risk. Compared to the *APOE* ε4 noncarrier, the risk of dementia among ε4 heterozygotes may be further amplified with regular PPI use. We speculated that PPIs might affect Aβ metabolism and synergize with the *APOE* ε4 to promote Aβ accumulation and increase dementia risk. PPIs may reduce lysosomal acidification by inhibiting V-ATPase activity, which is critical for Aβ clearance [25, 37].

Notably, the association between PPI use and dementia was not presented among *APOE* ε4 homozygotes. The *APOE* ε4 homozygote is a validated risk factor with significant effects, and its HRs of all-cause dementia and AD were 6.93 (95% CI, 6.05–7.92; *P* < 0.001) and 12.91 (95% CI, 10.59–15.75; *P* < 0.001) in this study. We hypothesized that the ε4 homozygotes are more likely with high loading of Aβ level, which may mask the relatively modest effects of PPI use by the mechanism like the epistasis effect [38]. When we investigated the combined effect of PPI use and *APOE* ε4, the results showed a significantly increased risk of dementia in ε4 homozygotes, regardless of whether they used PPI or not (Additional file 1: Fig. S2). In addition, PPIs may promote dementia by inducing vitamin B_12_ deficiency [39] or inhibiting choline acetyltransferase [40], but this has not been verified in this study.

Sex factors play an unavoidable role in the development of dementia. This study showed that the PPI-dementia association was more pronounced in females. Previous studies reported that females are more likely to develop dementia due to carrying *APOE* ε4 [41], which may be explained by the increased sensitivity of females to Aβ [42]. Thus, based on the hypothesis that PPIs promote dementia by increasing Aβ accumulation, we speculated that PPIs would synergize with the high Aβ sensitivity to increase the risk of dementia among females. In addition, the results of the subgroup analysis also suggested that the association between PPI use and dementia was more substantial in the non-smokers and participants without stroke. Smoking and stroke are often concomitant with cerebral oxidative stress and vascular inflammation, which are potential mechanisms for increased risk of AD [43]. Meanwhile, functional studies on primary human tissues and animal models showed PPIs had antioxidant and anti-inflammatory properties [44]. Therefore, we speculate that PPI use may neutralize the risk effect of smoking and stroke.

Our results showed that the association between different types of PPIs and dementia might differ, with lansoprazole being associated with dementia with greater strength than omeprazole at a relatively close statistical power. Consistent with earlier studies, results based on the AD cell model showed that the increase in Aβ levels after lansoprazole stimulation was more pronounced than omeprazole [29]. Lansoprazole also profoundly limits the retention of spatial information and the capacity to manipulate remembered memory to develop a strategy and execute a complex task [45]. In addition, there were more adverse effects of headaches after lansoprazole use [46]. Therefore, we believe that attention should be paid to the potential differences in PPIs in the nervous system.

Our study has several significant strengths, including the prospective population-based study design, the large sample size, and detailed information on related covariates, which provided adequate confounding adjustment and robust statistical power. In addition, individual genotype data set the stage for investigating drug-gene interactions. Thus, we demonstrated that PPI use and dementia associations might vary across *APOE* ε4 genotypes for the first time.

Some limitations should also be considered. First, PPI use was self-reported at baseline, and accurate dosage, duration, and validation by other sources were lacking. These may lead to recall bias and obscure within-group heterogeneity. This issue obstructed us from performing further analyses on these important factors. The primary exposure was based on data from a single baseline assessment only, and it cannot be excluded that a few participants only used the PPIs for a short period around the survey. Second, PPI use was not randomly assigned. Although we corrected for as many confounding factors and clinical indications as available, there may still be unmeasured confounding. Third, dementia consists of a complex set of symptomatic, and there may be diagnostic inaccuracies through ICD coding in electronic health records, while information on severity may be lost [47]. Due to the high under-diagnosis in the natural population, defining dementia based on hospital admissions and death registers may lead to missed diagnoses, and recorded dementia in these systems is often in an advanced stage. Besides, participants with comorbidities and prescription of PPI may have more contact with the health system and thus have a greater chance of being diagnosed with dementia. Fourth, considering the interpretability of the biological mechanisms, only one genetic risk factor, *APOE* ε4, was included in this study. In contrast, dementia and AD have complex genetic susceptibility factors, and the Aβ metabolism has complex regulatory mechanisms, and these may be the effect modifiers on the role of PPIs. Fifth, the UK Biobank study population may have intrinsic characteristics and limit the generalization of the results to other populations or nations.

## Conclusions

In conclusion, this population-based cohort study showed that regular PPI use was associated with an increased risk of incident all-cause dementia, AD, and VaD. Moreover, there was a significant interaction between PPI use and *APOE* ε4 genotype for dementia, and the association was most prominent in *APOE* ε4 heterozygotes. This study reveals prospective evidence and a potential mechanism for an association between PPI use and dementia, which requires further controlled trials and experimental studies to verify the causal relationship.

## Methods

### Study design

The UK Biobank study recruited more than 500,000 participants aged 40 to 70 years from the general population throughout the UK between 2006 and 2010 [48]. Participants provided information on health-related aspects through extensive baseline questionnaires, verbal interviews, and physical measurements. Participants were excluded if they withdrew from the study (*n* = 1298) and had prevalent dementia (*n* = 224). Then, we excluded 15,146 participants due to missing quality-controlled genotype data for subsequent analysis (Fig. 1).

### Ascertainment of exposure

The regular use of medications was collected through a verbal interview by a trained nurse at the baseline. “Regular” was defined as most days of the week for the past 4 weeks [49]. Data on short-term medication use, such as a 1-week course of antibiotics and medications they have recently stopped taking, were not recorded. Dosage and duration of medication use were not recorded in the UK Biobank. However, a repeat assessment conducted in 2012–2013 that included 20,346 participants showed 91.2% were consistent with their PPI use at baseline. PPIs mainly included omeprazole, esomeprazole, pantoprazole, lansoprazole, and rabeprazole. We combined the use of these drugs and defined regular PPI use as a dichotomous variable (yes or no).

### APOE genotyping

UK Biobank participants were genotyped using two genotyping arrays: UK BiLEVE or UK Biobank Axiom arrays. Following single nucleotide polymorphism (SNP) and sample quality controls, directly genotyped data were then imputed centrally by the UK Biobank based on the 1000 Genomes Phase 3, UK 10K haplotype, and Haplotype Reference Consortium reference panels [50]. *APOE* genotype was defined by two SNPs, rs429358 and rs7412. As *APOE* ε4 is a recognized genetic risk factor for dementia and AD mainly by affecting Aβ metabolism [31], we divided the population into *APOE* ε4 noncarriers (−/−), heterozygotes (+/−), and homozygotes (+/+) [51].

### Ascertainment of incident dementia

Data on defining dementia, including all-cause dementia, AD, and VaD, were obtained from the UK Biobank baseline assessment data, linked hospital admission data, and death register data. Diagnoses were recorded using the International Classification of Diseases (ICD) coding system (Additional file 1: Table S1) [52]. Participants with the incident disease were identified as having a primary and secondary diagnosis in hospital admission records or underlying and secondary causes of death from morbidity records post the date of baseline assessment. We calculated the follow-up time from the date of attendance until the date of first diagnosis, date of death, or February 25, 2018, for Wales and England, and February 28, 2017, for Scotland, whichever occurred first.

### Covariates

To control for potential confounding factors, we included the following covariates: sociodemographic characteristics (age, sex, ethnicity, education, household income, and Townsend deprivation index), lifestyle habits (smoking status, alcohol consumption, physical activity, body mass index [BMI], and occupational exposure), comorbidities (hypertension, coronary heart disease, diabetes, high cholesterol, stroke, traumatic brain injury, depression, anxiety, sleep apnea, cancer, GERD, Barrett’s esophagus, and gastroduodenal ulcer), and regular use of drugs or supplements (statin, antihypertensive drugs, anticholinergic drugs, benzodiazepines, z-hypnotics, aspirin, non-aspirin non-steroidal anti-inflammatory drugs [NSAIDs], multivitamin, and H2 receptor antagonists [H2RAs]). The Townsend deprivation index was used as an indicator of socioeconomic status and is provided directly by the UK Biobank [53]. Alcohol consumption was calculated based on the US Dietary Guidelines for Americans 2015–2020 [54]. Regular physical activity was calculated based on the validated International Physical Activity Questionnaire and categorized into three groups: regular, some, or no regular physical activity [55]. Information on medical history and use of drugs was collected via verbal interview at baseline. Anticholinergic drugs were defined by the Anticholinergic Cognitive Burden (ACB) scale and previous reports [56].

### Statistical analyses

Baseline characteristics of the participants were summarized across regular PPI users as numbers (percentage [%]) for categorical variables, mean (standard deviation [SD]) for normally distributed variables, or median (interquartile range) for skewed variables. The cumulative incident dementia outcomes were measured by the Kaplan-Meier method, and the differences between PPI users and PPI non-users were compared with the log-rank test. The analyses were conducted among the whole population and each *APOE* ε4 genotype group. To maximize the statistical power, we performed multiple imputations with chained equations (MICE) to assign missing covariate values. Detailed information on the number of missing covariates is shown in Additional file 1: Table S2.

The associations between regular use of PPIs and all-cause dementia, AD, and VaD outcomes were explored using Cox proportional hazard models with hazard ratios (HRs) and 95% confidence intervals (CIs). The assumption for proportional hazards was evaluated by tests based on Schoenfeld residuals [57], and violation of this assumption was not observed in our analyses. Three sets of models were performed. Model 1 was only adjusted for age and sex. Model 2 was adjusted for additional variables, including ethnicity, education, household income, Townsend deprivation index, smoking status, alcohol consumption, physical activity, BMI, occupational exposure, hypertension, coronary heart disease, diabetes, high cholesterol, stroke, traumatic brain injury, depression, anxiety, sleep apnea, cancer, and regular use of medications (statin, antihypertensive drugs, anticholinergic drugs, benzodiazepines, z-hypnotics, aspirin, non-aspirin NSAIDs, and multivitamin). To address the possible confounding effect of PPI use clinical indications, we additionally adjusted for GERD, Barrett’s esophagus, gastroduodenal ulcer, and regular H2RAs used in model 3.

To investigate potential effect modifiers, we conducted subgroup analyses according to *APOE* genotype (ε4 −/−, ε4 +/−, or ε4 +/+), sex (female or male), age (<65 or ≥65 years), obesity (BMI ≥ 30 kg/m^2^, yes or no), ever smoking (yes or no), ever drinking (alcohol consumption > 0, yes or no), hypertension (yes or no), stroke (yes or no), depression (yes or no), statin use (yes or no), and aspirin use (yes or no). The potential modifying effect was evaluated using the cross-product term of the stratifying variable with PPI use in the fully adjusted model.

We performed a series of sensitivity analyses. First, to reduce the influence of early-onset dementia, we conducted a sensitivity analysis of the associations among each *APOE* genotype after excluding participants under 65 years old at the baseline. Then, we performed sensitivity analyses by excluding participants who developed outcomes within 2 years to reduce potential reverse causations and excluding participants with missing values of covariates to validate the robustness of the results. Finally, we conducted a propensity score matching analysis to adjust the confounding factors with the matching ratio of 2:1. Propensity scores were estimated based on the multivariable logistic regression model by including all the covariates. All statistical analyses were performed using R v4.1.0 (R Center for Statistical Computing, Vienna, Austria), and statistical significance was determined at *P*-value < 0.05 (two-sided).

## Supplementary Information

**Additional file 1: Table S1.** Disease definitions used in the UK Biobank study. **Table S2.** The numbers (percentage) of the missing variables. **Table S3.** Subgroup analysis: associations of regular use of each PPI with the risk of incident dementia. **Table S4.** Sensitivity analysis: associations of regular PPI Use with the risk of incident dementia after excluding participants with missing covariate data. **Table S5.** Sensitivity analysis: associations of regular PPI use with the risk of incident dementia after excluding participants who developed outcomes during the first two years of follow-up. **Table S6.** Sensitivity analysis: associations of regular PPI use with the risk of incident dementia after excluding participants who developed outcomes only recorded on death register data. **Figure S1.** The cumulative risk of incident all-cause dementia (A), Alzheimer’s disease (B), and vascular dementia (C) according to regular PPI use for each APOE genotype subgroup. **Figure S2.** Association of regular PPI use and APOE genotype with incident dementia. **Figure S3.** Association of regular PPI use and modifying factors with incident all-cause dementia. **Figure S4.** Associations of regular PPI use with incident Alzheimer’s disease stratified by potential risk factors. **Figure S5.** Associations of regular PPI use with incident vascular dementia stratified by potential risk factors. **Figure S6.** Associations of regular PPI use with incident dementia stratified by APOE genotype among participants older than 65 years at baseline.

## Data Availability

Data are available in a public, open access repository. Data from the UK Biobank (https://www.ukbiobank.ac.uk/) are available to researchers on application.
