# Supplementary material for: Regular proton pump inhibitor use and incident dementia: population-based cohort study
Source: BMC Med. 2022 Sep 1;20:271. doi: 10.1186/s12916-022-02478-y (PMC9434890; doi:10.1186/s12916-022-02478-y)
Supplement: Supplementary file 1 — Additional file 1: Table S1. Disease definitions used in the UK Biobank study. Table S2. The numbers (percentage) of the missing variables. Table S3. Subgroup analysis: associations of regular use of each PPI with the risk of incident dementia. Table S4. Sensitivity analysis: associations of regular PPI Use with the risk of incident dementia after excluding participants with missing covariate data. Table S5. Sensitivity analysis: associations of regular PPI use with the risk of incident dementia after excluding participants who developed outcomes during the first two years of follow-up. Table S6. Sensitivity analysis: associations of regular PPI use with the risk of incident dementia after excluding participants who developed outcomes only recorded on death register data. Figure S1. The cumulative risk of incident all-cause dementia (A), Alzheimer’s disease (B), and vascular dementia (C) according to regular PPI use for each APOE genotype subgroup. Figure S2. Association of regular PPI use and APOE genotype with incident dementia. Figure S3. Association of regular PPI use and modifying factors with incident all-cause dementia. Figure S4. Associations of regular PPI use with incident Alzheimer’s disease stratified by potential risk factors. Figure S5. Associations of regular PPI use with incident vascular dementia stratified by potential risk factors. Figure S6. Associations of regular PPI use with incident dementia stratified by APOE genotype among participants older than 65 years at baseline. [file 12916_2022_2478_MOESM1_ESM.docx]

Additional file 1

Table of Contents

[Table S1. Disease definitions used in the UK Biobank study 1](#_Toc107567334)

[Table S2. The numbers (percentage) of the missing variables 2](#_Toc107567335)

[Table S3. Subgroup analysis: associations of regular use of each PPI with the risk of incident dementia 3](#_Toc107567336)

[Table S4. Sensitivity analysis: associations of regular PPI Use with the risk of incident dementia after excluding participants with missing covariate data 4](#_Toc107567337)

[Table S5. Sensitivity analysis: associations of regular PPI use with the risk of incident dementia after excluding participants who developed outcomes during the first two years of follow-up 5](#_Toc107567338)

[Table S6. Sensitivity analysis: associations of regular PPI use with the risk of incident dementia after excluding participants who developed outcomes only recorded on death register data 6](#_Toc107567339)

[Figure S1. The cumulative risk of incident all-cause dementia (A), Alzheimer’s disease (B), and vascular dementia (C) according to regular PPI use for each *APOE* genotype subgroup 7](#_Toc107567340)

[Figure S2. Association of regular PPI use and *APOE* genotype with incident dementia 8](#_Toc107567341)

[Figure S3. Association of regular PPI use and modifying factors with incident all-cause dementia 9](#_Toc107567342)

[Figure S4. Associations of regular PPI use with incident Alzheimer’s disease stratified by potential risk factors 10](#_Toc107567343)

[Figure S5. Associations of regular PPI use with incident vascular dementia stratified by potential risk factors 11](#_Toc107567344)

[Figure S6. Associations of regular PPI use with incident dementia stratified by *APOE* genotype among participants older than 65 years at baseline 12](#_Toc107567345)

# Table S1. Disease definitions used in the UK Biobank study

| Disease | UK Biobank Self Report Fields and Codes | ICD 9 | ICD 10 |
| --- | --- | --- | --- |
| All-cause dementia | Field 20002: Code 1263 | 290.2, 290.3, 290.4, 291.2, 294.1, 331.0, 331.1, 331.2, 331.5 | A81.0, F00, F00.0, F00.1, F00.2, F00.9, F01, F01.0, F01.1, F01.2, F01.3, F01.8, F01.9, F02, F02.0, F02.1, F02.2, F02.3, F02.4, F02.8, F03, F05.1, F10.6, G30, G30.0, G30.1, G30.8, G30.9, G31.0, G31.1, G31.8, I67.3 |
| Alzheimer's disease | - | 331.0 | F00, F00.0, F00.1, F00.2, F00.9, G30, G30.0, G30.1, G30.8, G30.9 |
| Vascular dementia | - | 290.4 | F01, F01.0, F01.1, F01.2, F01.3, F01.8, F01.9, I67.3 |

Abbreviations: ICD, International Classification of Diseases.

# Table S2. The numbers (percentage) of the missing variables

| Covariates | No. | % |
| --- | --- | --- |
| Household income | 76911 | 15.35 |
| Education | 10086 | 2.01 |
| Body mass index | 3061 | 0.61 |
| Smoking status | 2926 | 0.58 |
| Ethnicity | 2754 | 0.55 |
| Alcohol consumption | 1486 | 0.30 |
| Regular physical activity | 862 | 0.17 |
| Townsend deprivation index | 621 | 0.12 |

# Table S3. Subgroup analysis: associations of regular use of each PPI with the risk of incident dementia

| Outcomes | Non-users  (n = 447 267) | Omeprazole  (n = 33 125) | Model 3 ^a^ | | Lansoprazole  (n = 19 344) | Model 3 ^a^ | | Esomeprazole  (n = 1789) | Model 3 ^a^ | | Pantoprazole  (n = 972) | Model 3 ^a^ | | Rabeprazole  (n = 909) | Model 3 ^a^ | |
| --- | --- | --- | --- | --- | --- | --- | --- | --- | --- | --- | --- | --- | --- | --- | --- | --- |
|  | No. of Events (%) | No. of Events (%) | HR  (95% CI) | *P* | No. of Events (%) | HR  (95% CI) | *P* | No. of Events (%) | HR  (95% CI) | *P* | No. of Events (%) | HR  (95% CI) | *P* | No. of Events (%) | HR  (95% CI) | *P* |
| All-cause dementia | 5254 (1.17) | 736 (2.22) | 1.15 (1.00-1.32) | 0.056 | 471 (2.43) | 1.26 (1.07-1.48) | 0.007 | 50 (2.79) | 1.36 (0.87-2.13) | 0.173 | 27 (2.78) | 1.40 (0.76-2.57) | 0.281 | 24 (2.64) | 1.12 (0.60-2.10) | 0.729 |
| Alzheimer's disease | 2317 (0.52) | 300 (0.91) | 1.14 (0.90-1.44) | 0.274 | 186 (0.96) | 1.34 (1.02-1.76) | 0.036 | 21 (1.17) | 1.66 (0.84-3.31) | 0.147 | 13 (1.34) | 1.65 (0.67-4.11) | 0.279 | 6 (0.66) | 0.32 (0.04-2.27) | 0.252 |
| Vascular dementia | 1174 (0.26) | 223 (0.67) | 1.36 (1.04-1.80) | 0.027 | 150 (0.78) | 1.39 (1.01-1.91) | 0.046 | 10 (0.56) | 0.30 (0.04-2.13) | 0.226 | 6 (0.62) | 0.64 (0.09-4.66) | 0.662 | 13 (1.43) | 2.26 (0.91-5.59) | 0.079 |

Abbreviations: HR: hazard ratio; CI: confidence interval.

^a^ Estimated effects were based on the fully adjusted model.

# Table S4. Sensitivity analysis: associations of regular PPI Use with the risk of incident dementia after excluding participants with missing covariate data

| Outcomes | PPI Non-users (n = 345 606) | PPI Users (n = 39 244) | Model 3 ^a^ | |
| --- | --- | --- | --- | --- |
|  | No. of events (%) | No. of events (%) | HR (95% CI) | *P*-value |
| All-cause dementia | 1253 (0.36) | 313 (0.80) | 1.24 (1.07-1.43) | 0.004 |
| Alzheimer's disease | 469 (0.14) | 116 (0.30) | 1.34 (1.06-1.70) | 0.014 |
| Vascular dementia | 243 (0.07) | 82 (0.21) | 1.37 (1.02-1.84) | 0.035 |

Abbreviations: PPI, proton pump inhibitor; HR: hazard ratio; CI: confidence interval.

^a^ Estimated effects were based on the fully adjusted model.

# Table S5. Sensitivity analysis: associations of regular PPI use with the risk of incident dementia after excluding participants who developed outcomes during the first two years of follow-up

| Outcomes | PPI Non-users (n = 397 729) | PPI Users (n = 45 575) | Model 3 ^a^ | |
| --- | --- | --- | --- | --- |
|  | No. of events (%) | No. of events (%) | HR (95% CI) | *P*-value |
| All-cause dementia | 1685 (0.42) | 407 (0.89) | 1.20 (1.07-1.35) | 0.002 |
| Alzheimer's disease | 637 (0.16) | 152 (0.33) | 1.26 (1.04-1.53) | 0.017 |
| Vascular dementia | 329 (0.08) | 104 (0.23) | 1.24 (0.98-1.58) | 0.078 |

Abbreviations: PPI, proton pump inhibitor; HR: hazard ratio; CI: confidence interval.

^a^ Estimated effects were based on the fully adjusted model.

# Table S6. Sensitivity analysis: associations of regular PPI use with the risk of incident dementia after excluding participants who developed outcomes only recorded on death register data

| Outcomes | Total  Participants | PPI Non-users | PPI Users | Model 3 ^a^ | |
| --- | --- | --- | --- | --- | --- |
|  |  | No. of Events (%) | No. of Events (%) | HR (95% CI) | *P*-value |
| All-cause dementia | 500850 | 1880 (0.42) | 473 (0.89) | 1.20 (1.07-1.35) | 0.002 |
| Alzheimer's disease | 500949 | 707 (0.16) | 172 (0.32) | 1.24 (1.02-1.51) | 0.030 |
| Vascular dementia | 500968 | 367 (0.08) | 123 (0.23) | 1.26 (1.00-1.61) | 0.054 |

Abbreviations: PPI, proton pump inhibitor; HR: hazard ratio; CI: confidence interval.

^a^ Estimated effects were based on the fully adjusted model.

**
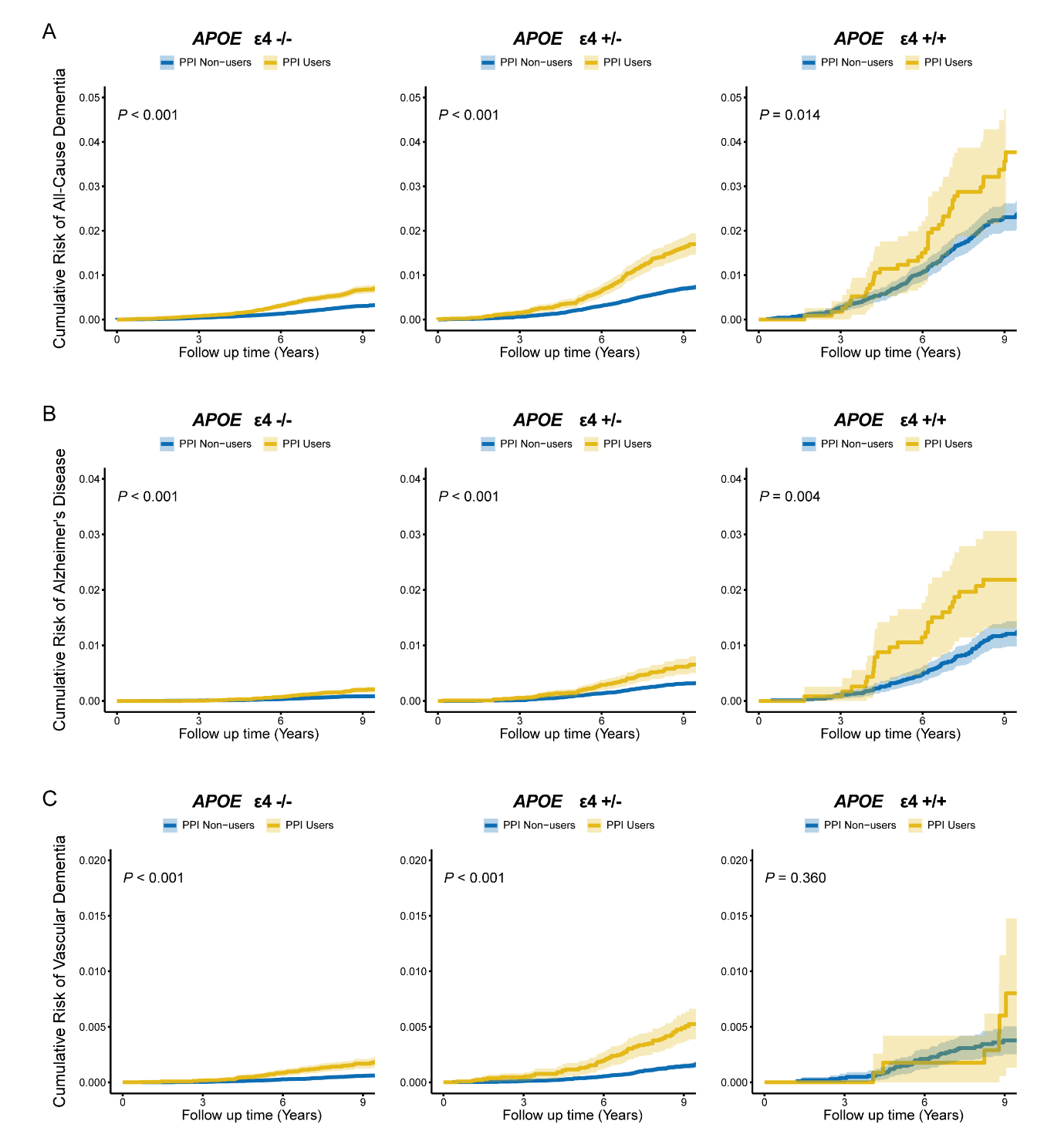
**

# Figure S1. The cumulative risk of incident all-cause dementia (A), Alzheimer’s disease (B), and vascular dementia (C) according to regular PPI use for each *APOE* genotype subgroup

Abbreviations: PPI, proton pump inhibitor; *APOE*, apolipoprotein E.


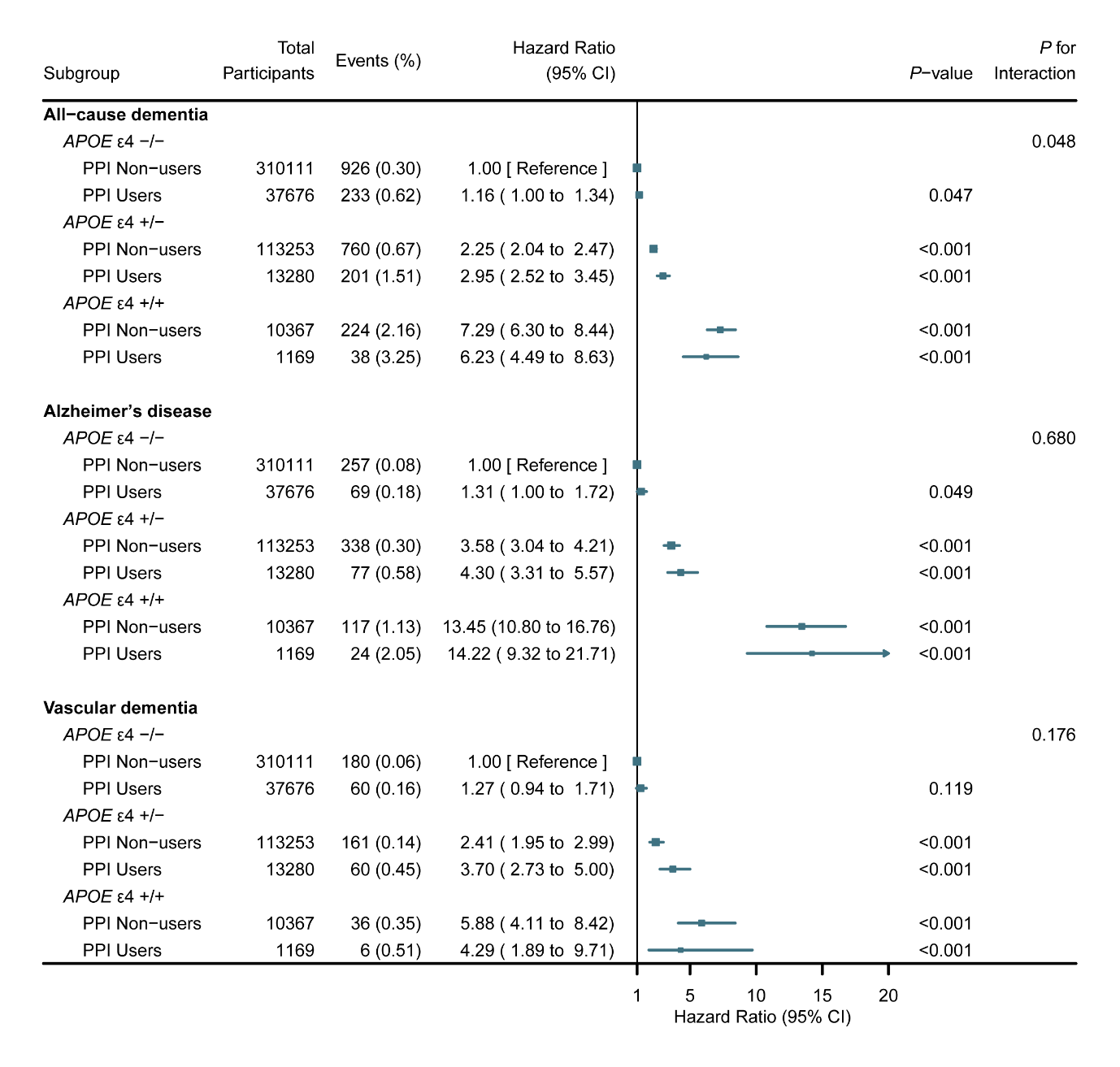


# Figure S2. Association of regular PPI use and *APOE* genotype with incident dementia

The vertical line indicates the reference value of 1. Estimated effects were based on the fully adjusted model.

Abbreviations: PPI, proton pump inhibitor; *APOE*, apolipoprotein E; HR, hazard ratio; CI, confidence interval.


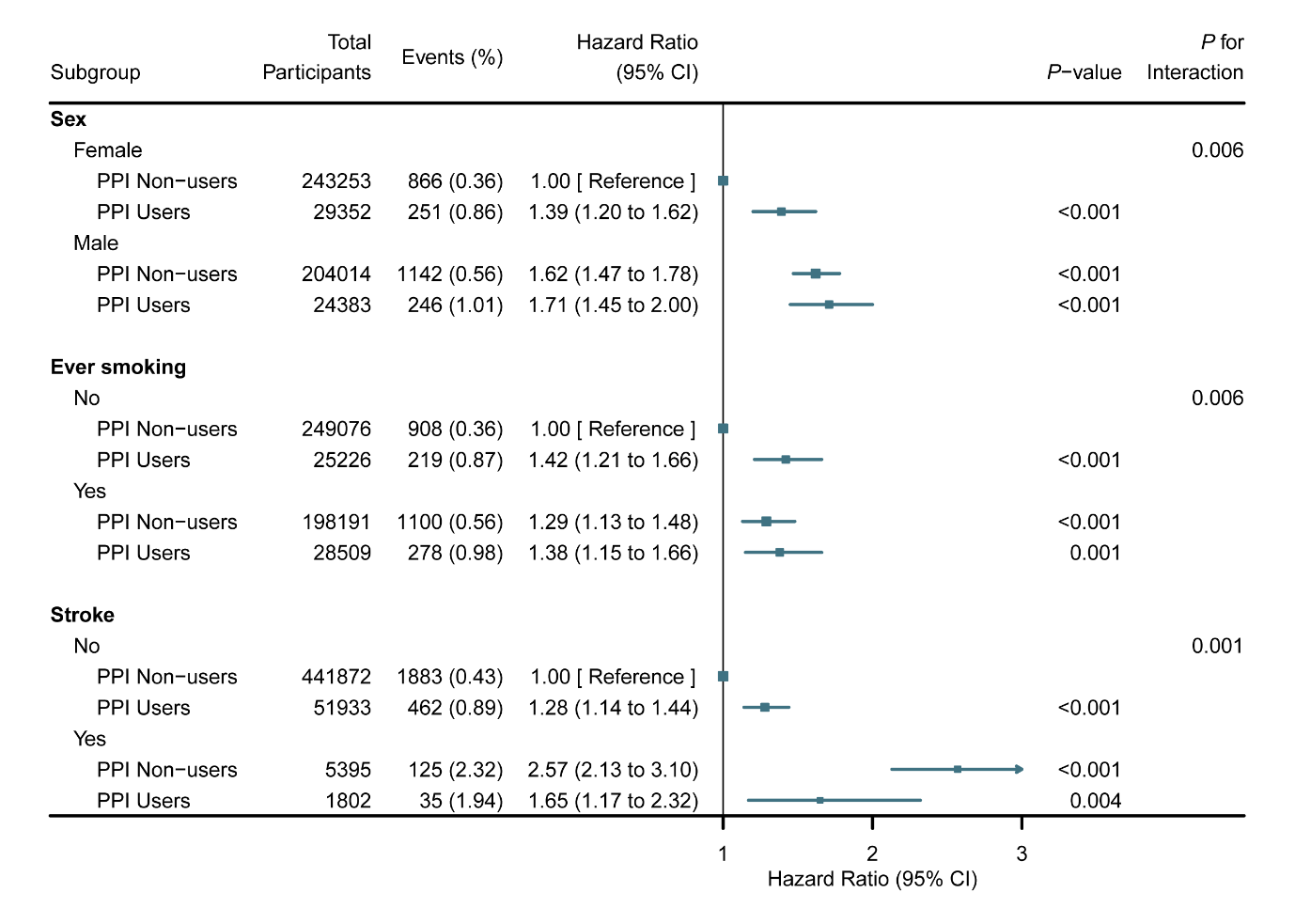


# Figure S3. Association of regular PPI use and modifying factors with incident all-cause dementia

The vertical line indicates the reference value of 1. Estimated effects were based on the fully adjusted model.

Abbreviations: PPI, proton pump inhibitor; HR, hazard ratio; CI, confidence interval.

**
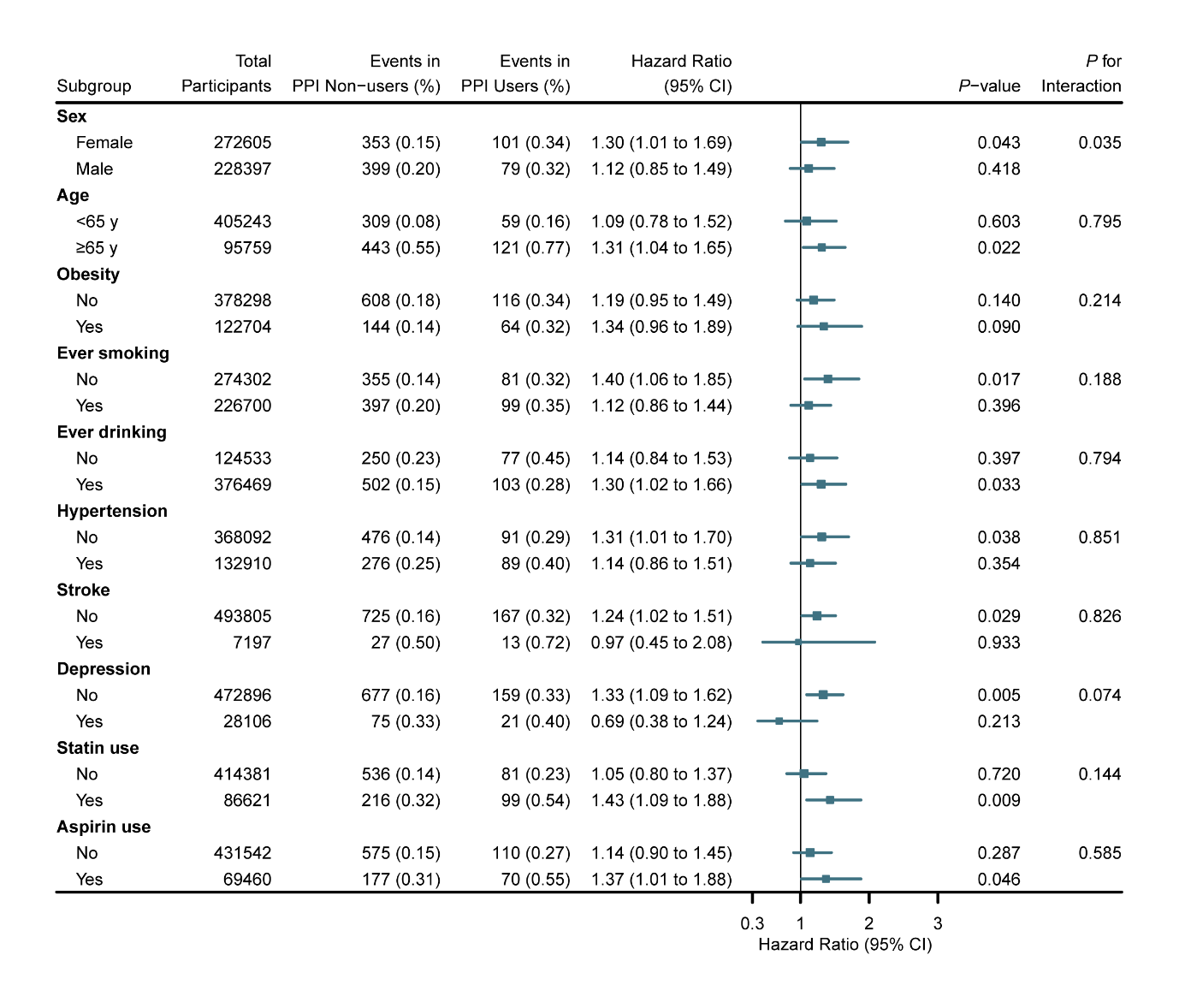
**

# Figure S4. Associations of regular PPI use with incident Alzheimer’s disease stratified by potential risk factors

The vertical line indicates the reference value of 1. Estimated effects were based on the fully adjusted model.

Abbreviations: PPI, proton pump inhibitor; HR, hazard ratio; CI, confidence interval.

**
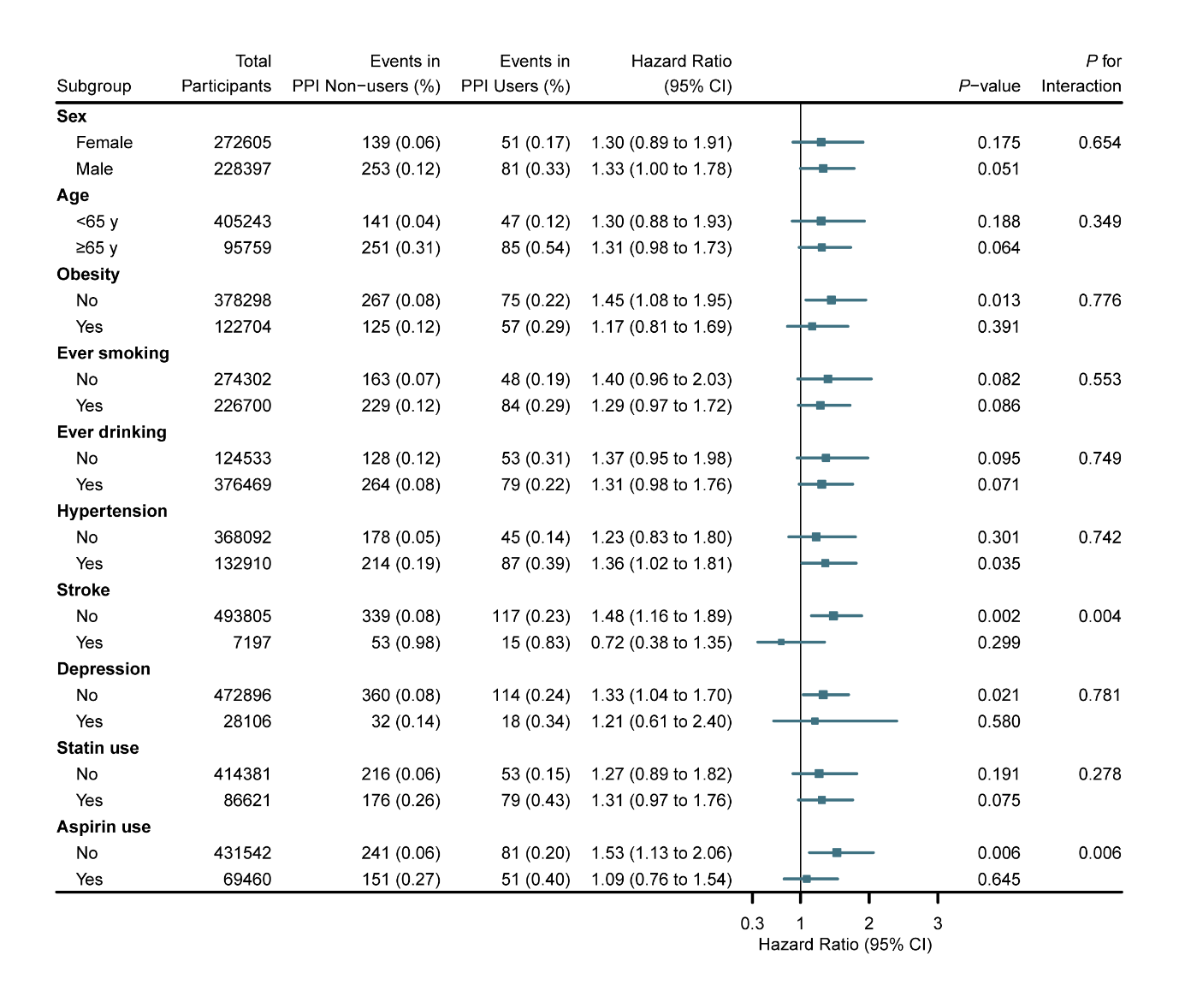
**

# Figure S5. Associations of regular PPI use with incident vascular dementia stratified by potential risk factors

The vertical line indicates the reference value of 1. Estimated effects were based on the fully adjusted model.

Abbreviations: PPI, proton pump inhibitor; HR, hazard ratio; CI, confidence interval.

**
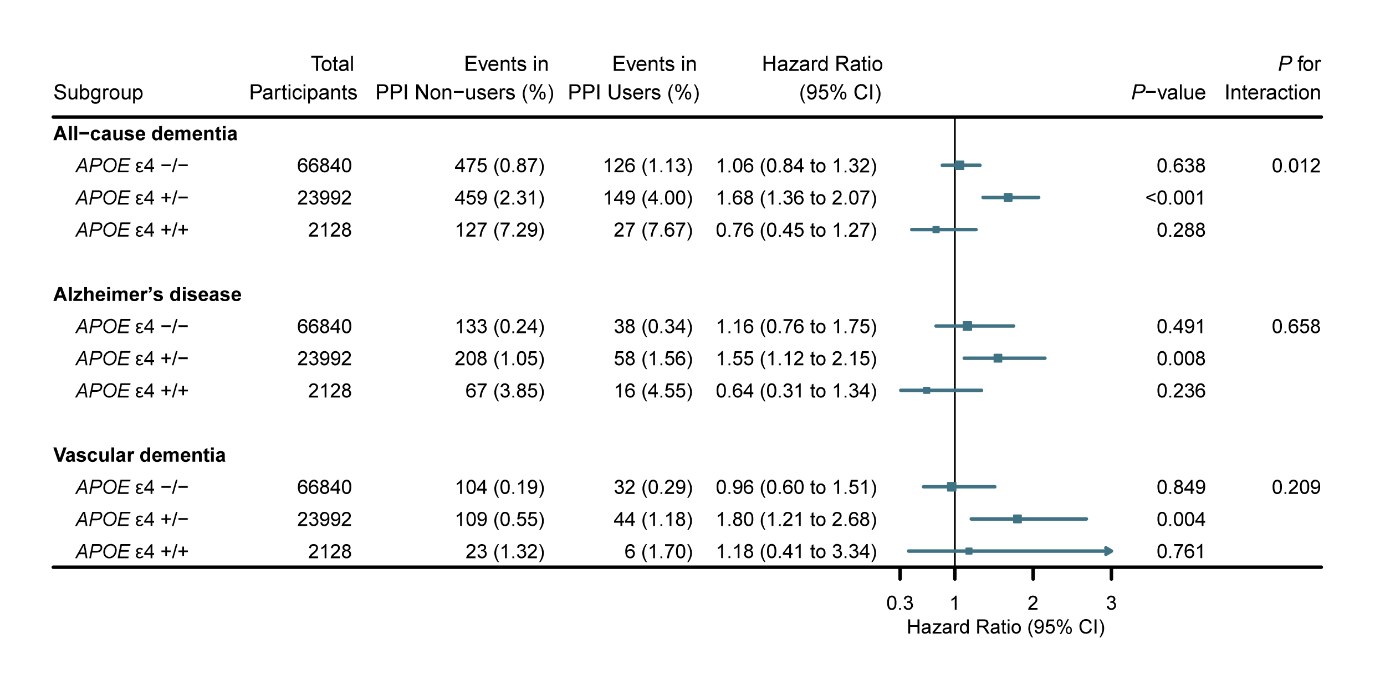
**

# Figure S6. Associations of regular PPI use with incident dementia stratified by *APOE* genotype among participants older than 65 years at baseline

The vertical line indicates the reference value of 1. Estimated effects were based on the fully adjusted model.

Abbreviations: PPI, proton pump inhibitor; *APOE*, apolipoprotein E; HR, hazard ratio; CI, confidence interval.
